# Supplementary material for: Red-Light-Only Control of Protein–Protein Interactions Using a Cyanobacteriochrome (UNICYCL)
Source: ACS Cent Sci. 2026 Jan 20;12(1):122–32. doi: 10.1021/acscentsci.5c01848 (PMC12856669; doi:10.1021/acscentsci.5c01848)
Supplement: Supplementary file 5 [file oc5c01848_si_005.pdf]

oc-2025-018483.R1

Name: Peer Review Information for "Red-light-only control of protein-protein interactions using a cyanobacteriochrome (UNICYCL)"

First Round of Reviewer Comments

Reviewer: 1

Comments to the Author

The manuscript by Le et al. describes the engineering of a light-dependent binding partner for CBCR GAF domain, a domain that undergoes a confomational change in red light. Notably, the partner is 6 kDa, forms a 1:1 complex in dark, and is dissociated with red light. This work nicely complements the other evolved light-dependent dimerizer tools available. Generally the manuscript is well written and quantitative, with conclusions supported by the data. The strength of the manuscript lies in the detailed structural characterization of the protein-based toolkit, with functional characterization adequate but more limited. The comparison of conformational changes of NpR6012g4 with those of PAS-GAF-PHY domains of D. radiodurans and plant PhyB are interesting. As there are few similar tools available (small domains that bind in dark but can be dissociated with red light) the reagents are likely to be useful and explored by the community for further engineering and application. The work is acceptable for publication, with several minor comments listed below.

1. A more in depth explanation of the constructs used to induce gene expression is warranted: Please describe what ‘E’ is (from what I could dig up from prior studies, it might be ‘erythromycin-responsive macrolide-inactivating 2’-phosphotransferase I repressor MphR(A)) but this should be included in the current paper. Also, what is ‘FUS N’ and what is its purpose in the construct?

2. “VP16 promoter transactivator” (Page 11, line 46)– assuming this refers to the activation domain of the VP16 protein of herpes simplex virus? If so, please specify.

3. In the schematic of Figure 2c, the VP16 piece seems to be bound to the minimal CMV promoter in dark- is this actually the case or would it be more accurate to show it ‘in proximity’ ?

4. Panels 2c, d: How long was light applied? (While this is in the methods, would be helpful to also have in figure legend).

Reviewer: 2

Comments to the Author

The authors report the design and characterization of a red-light sensitive photoswitch that can be used as an optogenetic tool. The switch is based on a minimum (GAF-only) cyanobacteriochrome that interconverts between a red- and a green-absorbing state by light. This CBCR fragment is a basis for the construction of a light-dependent complex with a small protein fragment (GA), with different (fluorescence) properties in the GA-bound and GA-free CBCR. The construct is tested in vitro and in vivo and structurally characterized by NMR. To make it brief: although I am not an expert in molecular biology/biochemistry, I am impressed by this study, the thoughtful design of the construct, the comprehensive analysis of the binding behavior, development and use of appropriate assays, and the structural characterization. Hence, in principle, the paper should be published as it is. However; the authors may consider revising the text a bit in view of the non-specialized readership of ACS Central Science. Specifically, it should be checked if all important expression/terms (e.g. GA) are sufficiently explained.

Author's Response to Peer Review Comments:

Reviewer(s)' Comments to Author:

Reviewer: 1

Recommendation: Publish in ACS Central Science after minor revisions noted.

Comments:

The manuscript by Le et al. describes the engineering of a light-dependent binding partner for CBCR GAF domain, a domain that undergoes a confomational change in red light. Notably, the partner is 6 kDa, forms a 1:1 complex in dark, and is dissociated with red light. This work nicely complements the other evolved light-dependent dimerizer tools available. Generally the manuscript is well written and quantitative, with conclusions supported by the data. The strength of the manuscript lies in the detailed structural characterization of the protein-based toolkit, with functional characterization adequate but more limited. The comparison of conformational changes of NpR6012g4 with those of PAS-GAF-PHY domains of D.
radiodurans and plant PhyB are interesting. As there are few similar tools available (small domains that bind in dark but can be dissociated with red light) the reagents are likely to be useful and explored by the community for further engineering and application. The work is acceptable for publication, with several minor comments listed below.

We thank the referee for the candid assessment and the positive comments on this work.

1. A more in depth explanation of the constructs used to induce gene expression is warranted: Please describe what 'E' is (from what I could dig up from prior studies, it might be 'erythromycin-responsive macrolide-inactivating 2'-phosphotransferase I repressor MphR(A)) but this should be included in the current paper. Also, what is 'FUS N' and what is its purpose in the construct?

We have added this information to the legend of Figure 2c "**E**, erythromycin repressor protein (MphR(A)); **(etr)$_8$**, eight etr repeat (E protein operator sequence); **FUS$_N$**, N-terminus (amino acids 1-214) of human oncogene FUS containing an intrinsically disordered region; **P$_{CMVmin}$**, minimal human cytomegalovirus immediate early promoter; **SEAP**, human

placental secreted alkaline phosphatase; **VP16**, Herpes simplex virus-derived transactivation domain."

2. "VP16 promoter transactivator" (Page 11, line 46)– assuming this refers to the activation domain of the VP16 protein of herpes simplex virus? If so, please specify.

We have specified VP16 as "Herpes simplex virus-derived transactivation domain VP16" on page 11.

3. In the schematic of Figure 2c, the VP16 piece seems to be bound to the minimal CMV promoter in dark- is this actually the case or would it be more accurate to show it 'in proximity' ?

Thank you for pointing this out. We have revised figure 2c so that VP16 is shown in proximity to the minimal CMV promoter, rather than appearing to bind to it.

4. Panels 2c, d: How long was light applied? (While this is in the methods, would be helpful to also have in figure legend).

We have added the following irradiation conditions to the legend of Figure 2d "Cells were illuminated for 24 h with 10 μmol m^{-2} s^{-1} light of 660 nm or kept in darkness prior to SEAP quantification."

Reviewer: 2

Recommendation: Publish in ACS Central Science without change.

Comments:

The authors report the design and characterization of a red-light sensitive photoswitch that can be used as an optogenetic tool. The switch is based on a minimum (GAF-only) cyanobacteriochrome that interconverts between a red- and a green-absorbing state by light. This CBCR fragment is a basis for the construction of a light-dependent complex with a small protein fragment (GA), with different (fluorescence) properties in the GA-bound and GA-free CBCR. The construct is tested in vitro and in vivo and structurally characterized by NMR. To make it brief: although I am not an expert in molecular biology/biochemistry, I am impressed by this study, the thoughtful design of the construct, the comprehensive analysis of the binding behavior, development and use of appropriate assays, and the structural characterization. Hence, in principle, the paper should be published as it is. However; the authors may consider revising the text a bit in view of the non-specialized readership of ACS Central Science. Specifically, it should be checked if all important expression/terms (e.g. GA) are sufficiently explained.

Thank you for your positive evaluation of this work!

We have revised the manuscript to provide brief descriptions of key terms (such as GA, CHO, VP16, etc.) at their first mention to ensure clarity for the non-specialized readership.

oc-2025-018483.R2

Name: Peer Review Information for "Red-light-only control of protein-protein interactions using a cyanobacteriochrome (UNICYCL)"

Second Round of Reviewer Comments

Reviewer: 1

Comments to the Author

The authors have suitably addressed all issues.

Reviewer: 2

Comments to the Author

All concerns were adequately considered in the revised version.

Author's Response to Peer Review Comments:

Please see requested corrections attached
